# Glucose-responsive hydrogel electrode for biocompatible glucose transistor

**DOI:** 10.1080/14686996.2016.1257344

**Published:** 2017-01-09

**Authors:** Taira Kajisa, Toshiya Sakata

**Affiliations:** ^a^ PROVIGATE Inc., Department of Research and Development, Tokyo, Japan; ^b^ Department of Materials Science and Engineering, Graduate School of Engineering, The University of Tokyo, Tokyo, Japan

**Keywords:** Field effect transistor, hydrogel, phenylboronic acid, glucose, biocompatibility, 30 Bio-inspired and, biomedical materials, 208 Sensors and actuators, 600 Others

## Abstract

In this paper, we propose a highly sensitive and biocompatible glucose sensor using a semiconductor-based field effect transistor (FET) with a functionalized hydrogel. The principle of the FET device contributes to the easy detection of ionic charges with high sensitivity, and the hydrogel coated on the electrode enables the specific detection of glucose with biocompatibility. The copolymerized hydrogel on the Au gate electrode of the FET device is optimized by controlling the mixture ratio of biocompatible 2-hydroxyethylmethacrylate (HEMA) as the main monomer and vinylphenylboronic acid (VPBA) as a glucose-responsive monomer. The gate surface potential of the hydrogel FETs shifts in the negative direction with increasing glucose concentration from 10 μM to 40 mM, which results from the increase in the negative charges on the basis of the diol-binding of PBA derivatives with glucose molecules in the hydrogel. Moreover, the hydrogel coated on the gate suppresses the signal noise caused by the nonspecific adsorption of proteins such as albumin. The hydrogel FET can serve as a highly sensitive and biocompatible glucose sensor in *in vivo* or *ex vivo* applications such as eye contact lenses and sheets adhering to the skin.

## Graphical abstract

**Figure:**
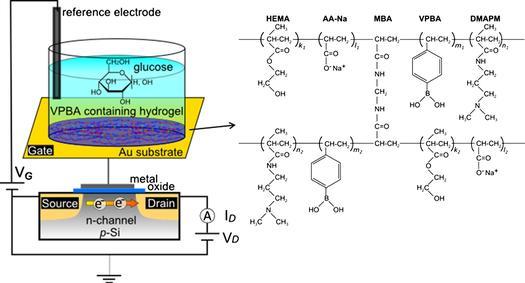

## 1. Introduction

Hydrogels are receiving considerable attention as biocompatible materials because of their flexibility. Since hydrogels behave as solid materials, retaining their shape even upon absorbing water, their physiological properties have contributed to the development of functional materials with a response to extrinsic stimuli such as pH and chemical substances.[1] In fact, hydrogel polymers are not only widely used as contact lenses owing to their water absorbability and moderate stiffness but are also applied as micro-micelle gels for drug delivery systems (DDSs).[2–4] Hydrogel polymers consist of hydrophilic monomers as the major component and are polymerized to form a three-dimensional cross-linked network, allowing functionalized monomers to be copolymerized depending on the purpose, based on chemical characteristics such as hydrophobicity/hydrophilicity, dissociation in response to a specific environment, and specific binding to target molecules. Therefore, hydrogels can be used as biocompatible biosensing electrodes to capture specific target molecules.

Enzyme electrodes can be used as glucose sensors [5] in a wide range of commercialized products such as for the self-monitoring of blood glucose (SMBG) to control the blood glucose level of diabetic patients. An enzyme such as glucose oxidase-glucose (GOX) or glucose dehydrogenase (GDH) is included in the polymer membrane on the electrode, in which glucose reacts with the enzyme resulting in a change in the redox potential. Such a device is miniaturized and convenient, and allows the detection of glucose levels even using a few μl of blood. However, diabetic patients require blood to be sampled for measurements at least a few times a day, such as before and after meals, which is troublesome and invasive. To solve the problems of a conventional glucose sensor, novel devices have been proposed that do not require blood sampling, which employ other principles such as infrared light.[6] Additionally, the glucose in biological fluids such as sweat, saliva, and tears can be measured instead of blood glucose,[7–9] although the glucose levels are approximately 1% of that in blood. To detect such dilute glucose, a highly sensitive and precise detection method is required in the miniaturized device.

**Scheme 1:**
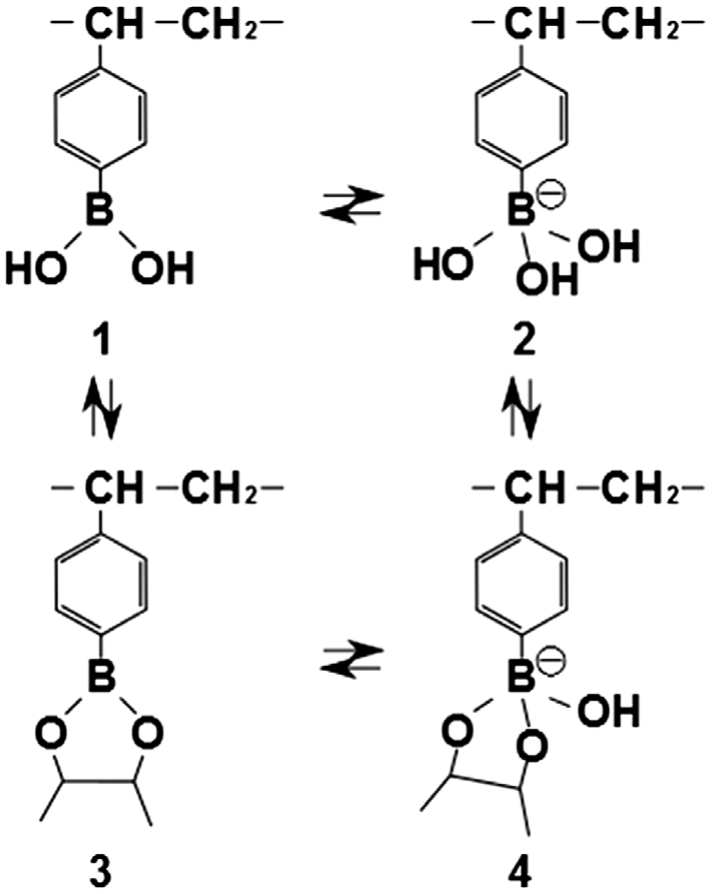
Equilibrium between vinylphenylboronic acid and a diol compound.

For this purpose, semiconductor technology may meet the requirements of a novel glucose sensor. Field effect transistor (FET) biosensors are suitable for the detection of small molecules such as glucose as long as they have molecular charges.[10,11] In particular, a FET biosensor combined with a phenylboronic acid (PBA) membrane showed good performance in detecting glucose at the μM level, enabling the detection of tear glucose with concentration of 10 μM order. PBA can reversibly bind to saccharides such as glucose and polyols by the ester binding of boronic acid and diol groups based on the charge equilibrium of boron, as shown in Scheme 1. Boron atoms, which form a non-ionic and electron-deficient state with a tertiary structure, are changed into anionic boron atoms with an electron-rich state having a quaternary structure when the diol group in a substance interacts with PBA.[12,13] Thus, a FET biosensor directly detects glucose from the change in the charge of PBA molecules caused by diol binding on the gate sensor surface. It is very important for practical use to enhance the signal to noise ratio for glucose sensing in biosamples including various impurities, but the strategy to prevent the effect of nonspecific adsorption on electrical signals has not been sufficiently considered for PBA-FET biosensors. Moreover, optimization of the biocompatibility for FET biosensors is required to realize future body-contactable biosensors that can directly detect the glucose in sweat, saliva, and tears.

In this study, we developed a glucose-responsive hydrogel electrode for FET biosensors. In particular, we coated a 2-hydroxyethylmethacrylate (HEMA) gel copolymerized with vinylphenylboronic acid (VPBA) on the gate electrode and investigated the suitability of its chemical and electrical properties for use in a hydrogel FET biosensor.

## 2. Experimental section

### 2.1. Materials

As the monomers constituting the hydrogel used in this study, which was a phenylboronic acid-containing gel, 2-hydroxyethylmethacrylate (HEMA, MW=130.14), 4-vinylphenylboronic acid (VPBA, MW=147.97), *N*-3-(dimethylamino) propylmethacrylamide (DMAPM, MW=170.25), and *N,N*-methylenebisacrylamide (MBA, MW=154.17) were obtained from Tokyo Chemical Industries Co., Ltd. (Tokyo, Japan), and used without further purification. Acrylic acid (AA, MW=72.06, Wako Pure Chemical Industries Ltd, Japan) was neutralized to pH 7.4 with 1 M NaOH and used as 6.7 wt% AA solution. The copolymerization was initiated with a potassium persulfate (KPS, Wako) and tetramethylethylenediamine (TEMED, Wako). Ultrapure water (Komatsu Electronics Co., Ltd., Ishikawa, Japan) was used in all experiments.

### 2.2. Preparation of Au substrate

An Au film with a thickness of approximately 100 nm and then a Ti layer with a thickness of approximately 15 nm were sputtered on a white cut glass slide (Matsunami Glass Ind., Ltd., Osaka, Japan). A transparent polycarbonate ring (18 mm inner diameter/20 mm outer diameter) was encapsulated on the Au substrate using an epoxy resin (ZC-203T; Pelnox Ltd., Kanagawa, Japan) except for the sensing surface. The Au sensing surface was treated with a UV ozone cleaner (Meiwafosis Co., Ltd., Tokyo, Japan) to remove organic compounds before the copolymerization of the hydrogel.

### 2.3. Copolymerization of hydrogel matrix

The hydrogel was obtained by the copolymerization of HEMA and VPBA monomers in accordance with the study of Özdemir and Tuncel with slight modification.[14] The main-chain monomers (HEMA and DMAPM) with 30 wt% solid content and the hydrogel with 3.0 wt% of VPBA content were prepared as follows: HEMA (0.40 g), DMAPM (0.20 g), VPBA (0.06 g), MBA (0.01 g), and 6.7 wt% AA solution (1.2 g) were diluted with deionized water to form solutions with a weight of 2.0 g. Monomer solution of each hydrogel with 10 or 15 wt% solid content and 0 to 1.5 wt% VPBA content were prepared by varying the monomer ratio and adjusting to the weight to 2.0 g for each solution. Each diluted solution was sonicated for 5 min and degassed for 10 min in vacuum. As polymerization initiators, 10 μl of TEMED and 80 μl of KPS solution (50 mg ml^–1^) were added to the monomer solution, and were mixed on ice. The monomer solution (20 μl) was added by pipette to the Au surface, and a polyethylene terephthalate (PET) coverslip was placed on the monomer solution without the formation of air bubbles. To observe the effect of albumin inhibition, 200 μl of the monomer solution was added to the Au surface to fabricate a thicker hydrogel. The gel was formed at room temperature for 4 h under a nitrogen atmosphere. After gel formation, the coverslip was gently peeled off and the gel was equilibrated by immersion in phosphate-buffered saline (PBS) solution (pH 7.4) overnight.

### 2.4. Characterization of hydrogels

The swelling ratio of the prepared hydrogels in glucose media at room temperature was determined. The fabricated gels were swelled in PBS buffer (pH 7.4) including 100 mM glucose at room temperature for seven days. The weight of the swollen gel (W_s_) was measured with an electronic balance to 0.01 g after removing the excess surface glucose solution by a filter paper. The swollen gel samples were dried at 70 °C for three days, then the weight of the dried gel (W_d_) was measured. The equilibrium swelling ratio was calculated as [14]:

$${\text{Q = (W}}_{\text{s}}-{\text{W}}_{\text{d}}\text{)}\;\text{/}\;{\text{W}}_{\text{d}}$$

### 2.5. Real-time and electrical monitoring of glucose response using hydrogel gate FET

The electrical properties of the hydrogel FET biosensor were measured as a function of the glucose concentration in real time. PBS buffer (1.5 ml, pH 7.4) was poured into the gel-based Au gate equipped with a 20-mm-diameter polycarbonate ring and equilibrated until the gate surface voltage was stabilized. After the stabilization of the surface voltage in the PBS buffer, glucose solution was gradually added to a total concentration of 10 μM to 40 mM. The time course of the surface potential at the Au gate surface was monitored using a FET real-time monitoring system (Optgenesys Co., Ltd, Saitama, Japan). The change in the surface potential was monitored at a constant drain voltage of 2 V and a constant drain-source current of 700 μA.

## 3. Results and discussion

### 3.1. Optimization of copolymerized hydrogel

**Figure 1.**
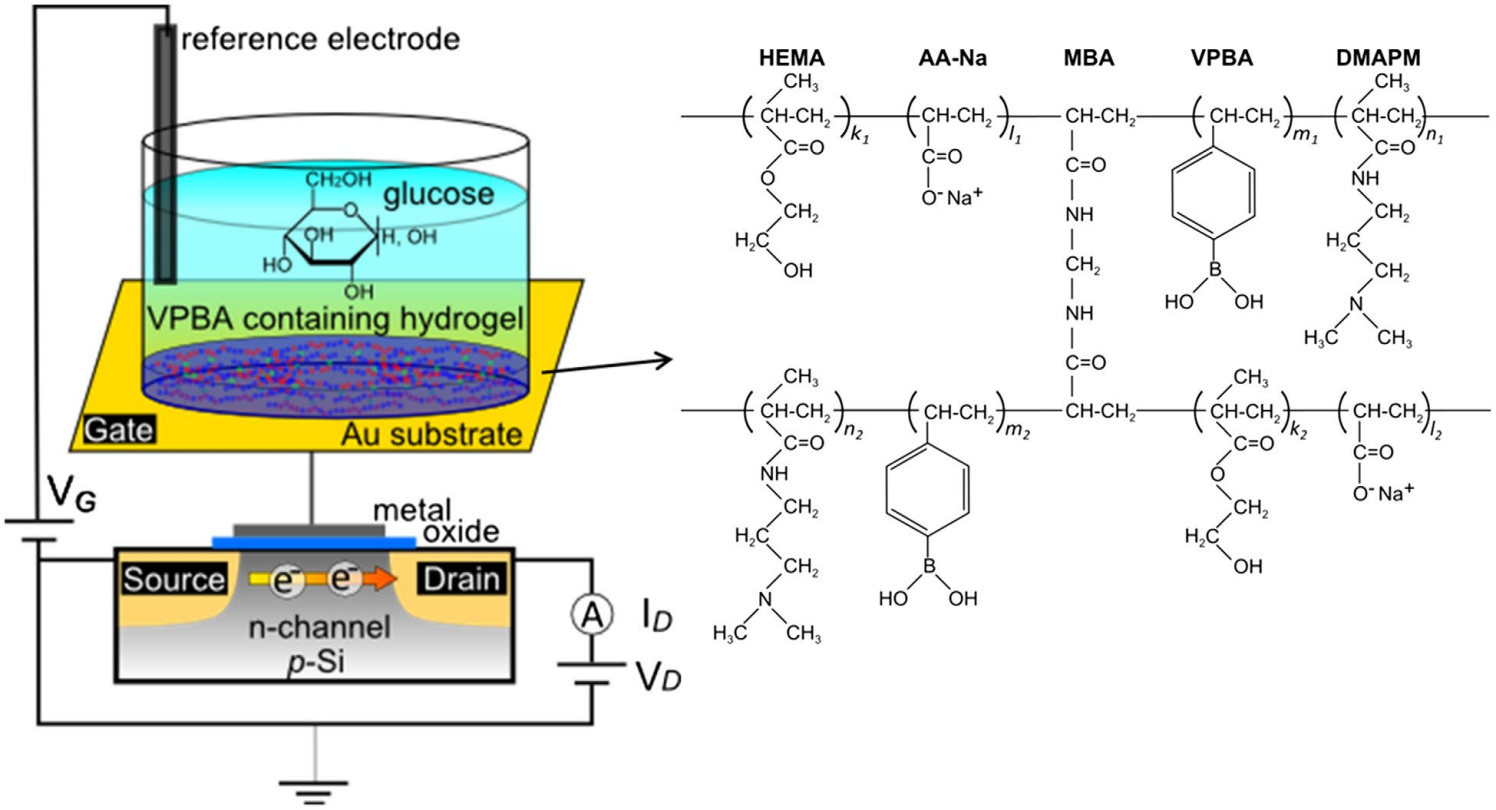
Schematic illustration of copolymerized hydrogel-based FET for glucose recognition. The FET was composed of an extended-gate FET with an Au substrate. A thin layer of hydrogel with a structural formula of poly(HEMA-*ran*-DMAPM-*ran*-VPBA-*ran*-AA) was copolymerized on the Au surface, where it interacted with glucose. HEMA and DMAPM were used as the main-chain monomers, AA was used to improve absorbability and VPBA was used as the glucose recognition component.

Figure 1 shows a schematic illustration of the glucose-responsive hydrogel-coated gate FET (hydrogel FET) and the chemical structure of the hydrogel used in this study. The hydrogel FET is sensitive to changes in the charge of VPBA bound with glucose in the hydrogel coated on the Au gate surface. As shown in Scheme 1, the VPBA in the hydrogel is initially in the equilibrium state between uncharged (states 1, 3) and anionic charged (states 2, 4) forms. The equilibrium of VPBA shifts from an uncharged state (state 1) to an anionic charged state (state 4) upon the addition of diol compounds such as glucose. A homogeneously polymerized hydrogel should be deposited on the electrode to quantitatively detect the glucose concentration. To achieve it, the amount of the copolymerized monomers was controlled in each prepared hydrogel. HEMA and DMAPM were utilized as the solid content, whose total concentration was set to 30, 15, or 10 wt%. The VPBA content was 0, 0.5, 1.5, or 3.0 wt% for each gel. Poly-HEMA, which was used as the main monomer in this study, is widely used as a hydrophilic and biocompatible material, and is generally known to be safe to attach directly to the body surface.[15] DMAPM was used to introduce amino groups into the phenylborate copolymers to enhance the binding ability of VPBA with glucose. This is why cationic amino groups in DMAPM are likely to induce anionic PBA on the basis of an electrostatic interaction, resulting in the enhanced responsibility of VPBA in the hydrogel to glucose, as shown in Scheme 1. This is effective for ensuring a response to glucose at physiological pH, because p*Ka* for PBA is 8.8.[16–19] AA was copolymerized to improve its water absorbability because it is generally used as a superabsorbent polymer (SAP).

**Figure 2.**
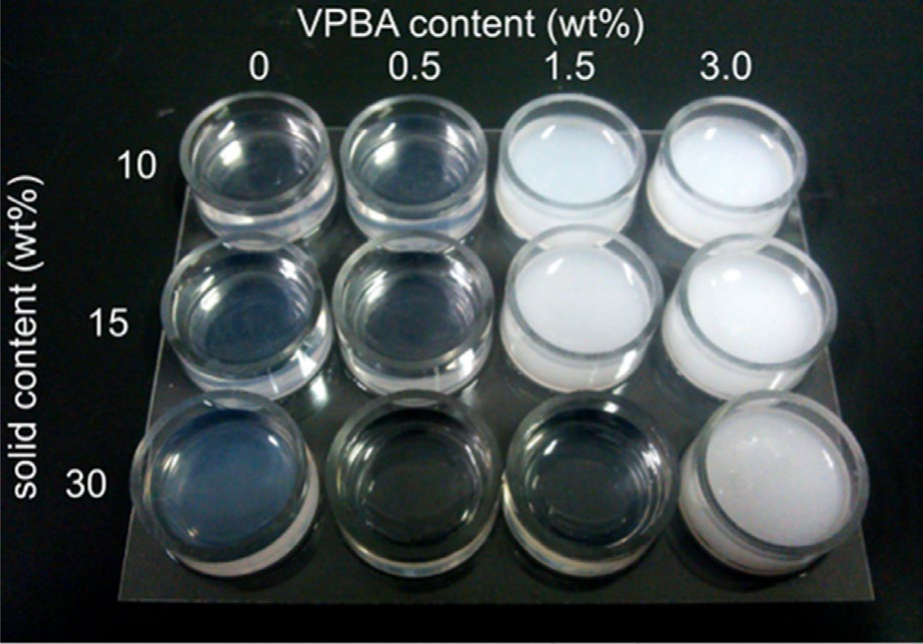
Photograph of hydrogels copolymerized with different mixing ratios of the solid content and VPBA contents. The solid content of the monomers was changed from 10 to 30 wt%, as shown in the vertical direction, and the VPBA content was changed from 0 to 3 wt%, as shown in the horizontal direction.

**Figure 3.**
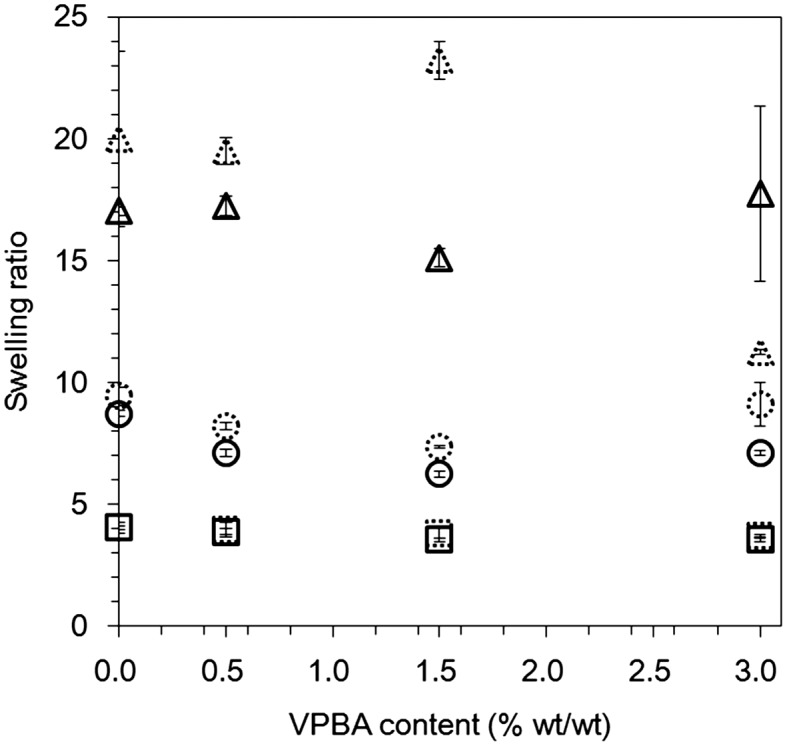
Swelling ratios of hydrogels copolymerized with different mixing ratios of the solid content and VPBA contents. The hydrogels were immersed in the buffer solution with or without glucose. The dotted line indicates the swelling ratio after equilibration with water, and the solid line indicates the swelling ratio after equilibration with 100 mM glucose. (Triangles: 10 wt% solid content, circles: 15 wt% solid content, squares: 30 wt% solid content).

**Figure 4.**
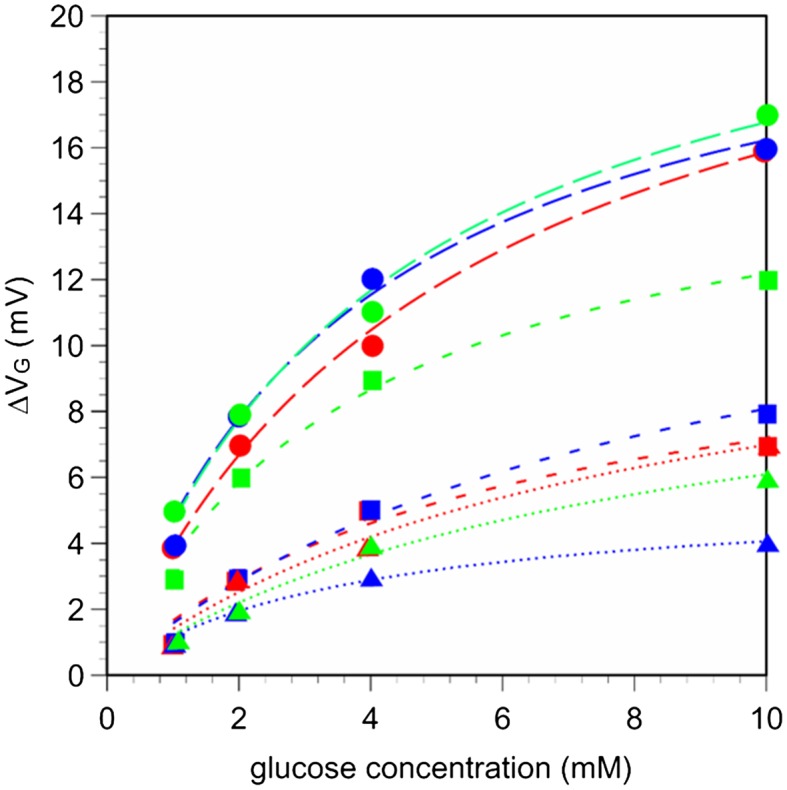
Shifts in gate voltage in hydrogel FETs with different mixing ratios of the solid content and VPBA contents upon a change in the concentration of glucose. (Squares: 10 wt% solid content, circles: 15 wt% solid content, triangles: 30 wt% solid content; Red, blue, and green show VPBA concentrations of 0.5, 1.5, and 3.0 wt%, respectively.)

**Figure 5.**
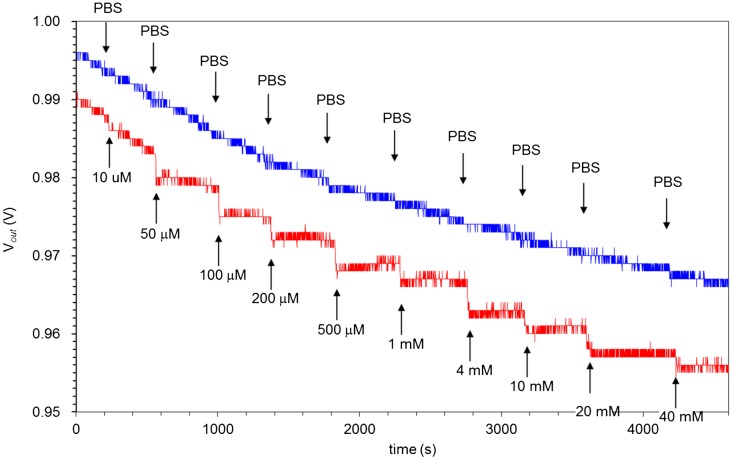
Real-time changes in surface potential of 15/0.5 hydrogel FET upon the addition of glucose in the range of 10 μM to 40 mM at room temperature. At the arrows, each sample solution was introduced onto the 15/0.5 hydrogel FET. The blue line shows the signal obtained with the control sensor, in which PBS buffer solutions without glucose were added, while the red line shows the signal obtained with the 15/0.5 hydrogel FET, in which PBS buffer solutions with glucose were added.

**Figure 6.**
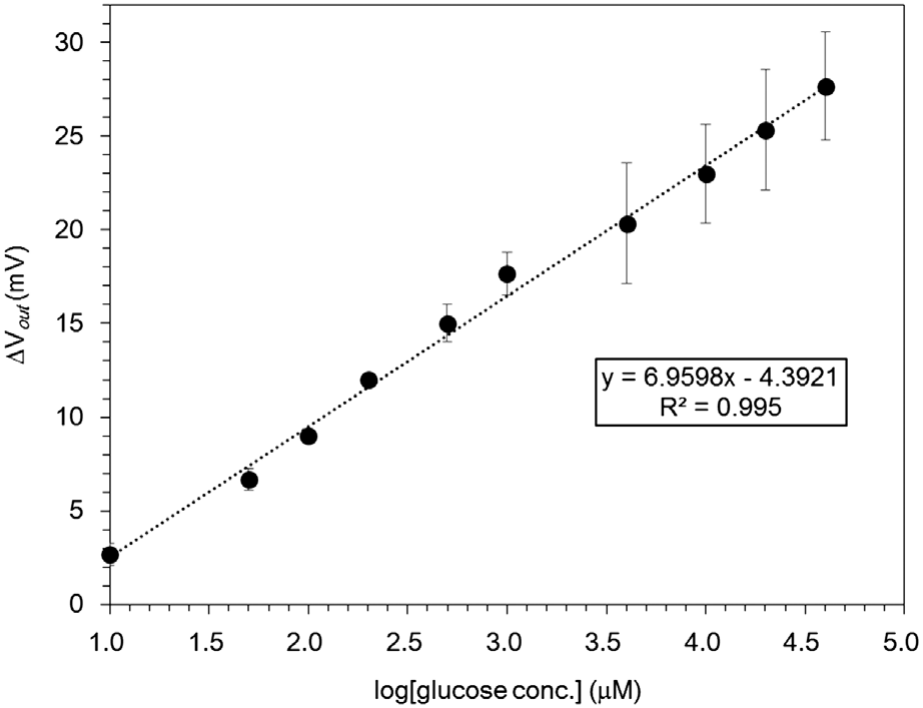
Changes in the surface potential of 15/0.5 hydrogel FET with logarithm of glucose concentration based on the signals shown in Figure 5. The linear fit corresponds to R^2^ = 0.995.

**Figure 7.**
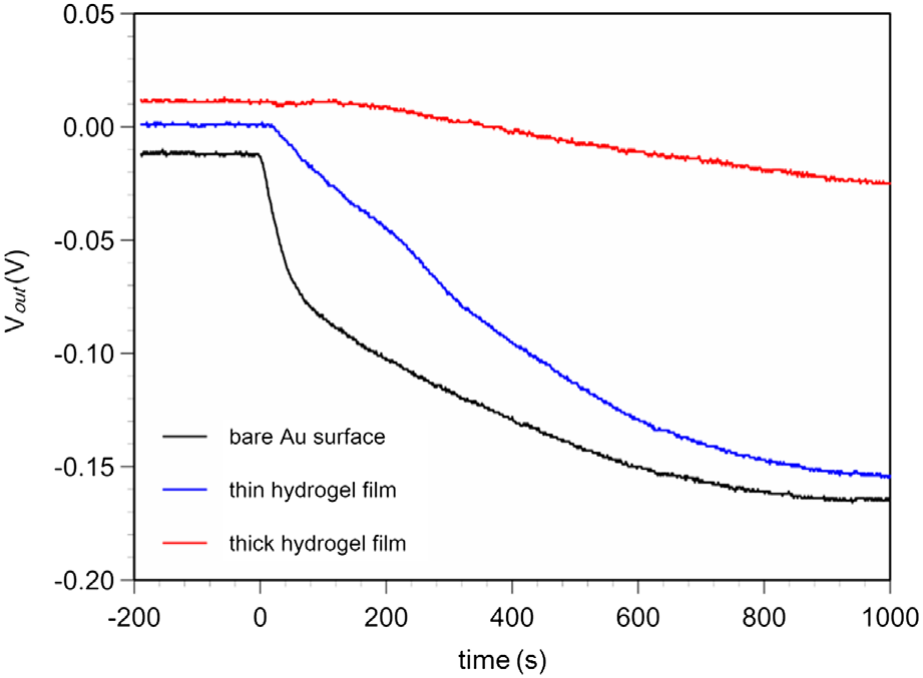
Real-time monitoring of surface potential with hydrogel FETs with different hydrogel thicknesses. (Black: unmodified Au gate, blue: hydrogel-modified Au gate copolymerized by 20 μl monomer solution, red: hydrogel-modified Au gate copolymerized by 200 μl monomer solution.)

The morphological properties of the poly(HEMA-*ran*-DMAPM-*ran*-VPBA-*ran*-AA) hydrogel were observed during its phase separation in the polymerization process, as shown in Figure 2. Regardless of the solid content, the gel with 0 and 0.5 wt% of VPBA were transparent or semi-transparent after polymerization, while the hydrogels became white as the VPBA content increased. This indicates that phase separation occurred in the process of polymerization. Thus, the phase separation was caused by the mixing ratio of monomers with different chemical properties and water contents. Since the aromatic VPBA monomer is basically immiscible in a solution because of its hydrophobicity, the hydrogel is likely to be cloudy with increasing content of VPBA solution or rate of water solution (which corresponds to a decrease in the solid contents). In addition, the phase equilibrium behavior of multicomponent monomers follows Flory–Huggins theory due to the lattice model according to microphase separation.[20,21] Therefore, the morphological changes in the hydrogels shown in Figure 2 resulted from some of the factors affecting microphase separation such as the solute–solvent ratio and the mixing ratio used in the copolymerization of the multi-component monomers. Taking into consideration the factors mentioned above, the gels with 0.5 wt% VPBA appear to be suitable for use with the electrode, regardless of the solid content, because these hydrogels were uniformly copolymerized without the aggregation of monomers, which should contribute to the quantitative detection of signals, although morphological homogeneity was also observed for the gel including 30 wt% solid content and 1.5 wt% VPBA. As an example of the morphology of hydrogels, which contained 15 wt% solid content and 0.5 wt% VPBA, Figure S1 (Supplemental material) shows that the hydrogel thickness was within 10 μm and the gel adhered tightly on the Au gate surface.

To investigate the effect of glucose addition on the swelling of the hydrogels, the prepared hydrogels were immersed in 100 mM glucose solution for seven days (Figure 3). Regardless of whether or not glucose was included in the solution, the swelling ratios of the hydrogels with 30 wt% solid content remained relatively unchanged regardless of the VPBA content. This property was less distinct for the hydrogels with 10 wt% solid content. Moreover, the swelling ratios of the hydrogels with 15 wt% solid content changed with the VPBA contents. That is, the swelling ratio was relatively large for the 3.0 wt% VPBA hydrogel, while it was relatively small for the 0, 0.5, and 1.5 wt% VPBA hydrogels. The swelling/deswelling of the hydrogel on the gate causes a change in the permittivity, resulting in the voltage shift of FET. This electrical property of the hydrogel should neutralize the electrical signal based on the change in the charge of PBA induced by glucose (see Section 3.2). Therefore, a clear response of the hydrogel FET to glucose can be expected for all hydrogels with 0.5 wt% VPBA and the hydrogel with 30 wt% solid content and 1.5 wt% VPBA, considering the phase separation and the change in permittivity of the hydrogels.

### 3.2. Potential response of FET biosensor with VPBA hydrogel for glucose recognition

The electrical properties of hydrogel FETs were approximately evaluated to assess the ability of the hydrogels to efficiently detect signals with high sensitivity. The changes in the gate voltage changes for each hydrogel FET are plotted in Figure 4. The hydrogel FETs with 15 wt% solid content produced larger electrical signals. Among the hydrogels discussed in Section 3.1, the 15 wt% HEMA/DMAPM and 0.5 wt% VPBA hydrogel (15/0.5 hydrogel) can be utilized as a representative electrode with a homogeneous hydrogel. Therefore, the electrical signals of the 15/0.5 hydrogel FET were examined as a function of glucose concentration using a real-time monitoring system, as shown in Figure 5. The time course of V_*out*_ for the diol binding of glucose with VPBA-copolymerized gel in the PBS buffer solution (pH 7.4) was monitored. The final concentration of glucose was increased from 10 μM to 40 mM in a stepwise manner. V_*out*_ shifted in the negative direction for every concentration to form a staircase pattern, while it did not shift for the control sensor, on which the PBS buffer without glucose was added. This is because the PBA groups in the gel were negatively charged by the equilibrium shift to the quaternary state (state 4 in Scheme 1) as a result of the diol binding on the Au gate electrode, although the detected signal might have been slightly affected by the swelling. If the swelling of the hydrogel had been detected with this sensor, V_*out*_ would have shifted in the positive direction due to the increase in the capacitance of the hydrogel resulting from its hydration. Actually, the swelling ratio for the hydrogel used in this measurement was relatively small regardless of whether or not glucose was added (Figure 3). Moreover, the shifts of the signal in the negative direction were larger than those for the swelled hydrogel FETs, as shown in Figure 4. That is, the hydration of the 15/0.5 hydrogel was mainly caused by not the hydration of the HEMA component itself, but by the ionic change in the boron atom. Therefore, the 15/0.5 hydrogel FET biosensor was able to detect a change in the ionic charges in PBA based on diol binding with glucose, regardless of the change in capacitance.

Figure 6 shows the relationship between the surface potential shift (ΔV) and the logarithm of the glucose concentration in the range of 10 μM to 40 mM based on the data shown in Figure 5. This experiment was repeated at least three times. The concentration of glucose can be estimated quantitatively using the hydrogel FET from the shift in the surface potential. The response to glucose was about 7.0 mV per decade of glucose concentration, which was similar to that for an Au gate FET coated with mercaptophenylboronic acid (MPBA; a self-assembled monolayer (SAM)) of ca. 8.4 mV/decade of glucose concentration between 50 μM and 20 mM in a previous work.[11] La Belle et al. [22] calculated the limited lowest detection (LLD) of glucose to be 43.4 μM in the range of 200 μM to 1 mM for an enzyme electrode combined with a microfluidic system^[22]^, while the detection limit in this study was calculated to be about 5 μM from the semi-logarithmic plots in the range of 10 μM to 40 mM shown in Figure 6, resulting from the Kaiser limit theory.[23]

The blood glucose level is approximately 5 mM for healthy persons, while that for diabetic patients is lower (2–3 mM) or higher (over 10 mM), resulting in inappropriate insulin release. On the other hand, the glucose level in biological fluids is approximately 1% of that in the blood. Moreover, as shown in Figure 6, the VPBA/hydrogel FET can detect glucose at concentrations of less than 1 mM, the standard deviations of which were within 5%, although higher glucose levels caused larger variations of ΔV_out_. Therefore, the VPBA/hydrogel FET can detect glucose in the biological fluids of diabetic patients with low or high glucose levels, although our device may be inadequate for the detection of blood glucose in diabetic patients with high glucose levels. However, the detection sensitivity of the VPBA/hydrogel FET was relatively low (about 7 mV/decade); thus, it should be improved because the enhancement of detection sensitivity contributes to the discrimination between glucose and other impurities in biological fluids.

### 3.3. Inhibition of albumin adsorption on gold surface by hydrogel

Not only blood but also body fluids contain various proteins such as lysozyme, lactoferrin, immunoglobulin, and albumin.[24,25] In particular, it was reported that albumin can easily bind to gold by -S-Au binding because it has cystein and disulfide bonds at the protein surface.[26–28] When a gold electrode is used as the gate of a FET biosensor, it is important to remove the effect of albumin adsorption on the surface. To achieve this, the HEMA/VPBA-copolymerized hydrogel membrane on the gold electrode has a molecular sieve effect that prevents protein adsorption as well as inducing the specific detection of glucose. Hydrogels with different thicknesses were copolymerized by immersing the gold electrode in the glass ring in 20 μl of monomer solution (thin film) or 200 μl of monomer solution (thick film), while an unmodified gold electrode was prepared as a negative control sensor. Albumin with 1 g l^–1^ was added to the measurement solution. Figure 7 shows a time course for the change in surface potential due to albumin addition for each sensor. First, the surface potential immediately decreased for the unmodified Au gate FET. In this case, a shift of about 150 mV was observed because albumin can easily bind to Au. In the hydrogel FET coated with 20 μl of monomer solution, the shift in the surface potential was slightly delayed until about 20 s after albumin addition, and the rate of decrease was relatively moderate compared with that for the unmodified Au gate FET, even though the final shift after 1000 s was about 150 mV, the same as that of the unmodified Au gate FET. On the other hand, the albumin adsorption was significantly suppressed for the hydrogel FET, which was copolymerized by 200 μl of monomer solution. In this case, the surface potential began to decrease about 200 s after albumin addition, and the total shift was about 30 mV after 1000 s. In this study, the hydrophilic hydrogel film on the gold gate electrode prevented the drift of the signal due to albumin adsorption on the gold surface. The HEMA in the prepared hydrogel had the ability to eliminate albumin adsorption by utilizing the chemical properties of hydroxyl groups.[29,30] Therefore, the HEMA/VPBA hydrogel FET sensor is expected to be useful for glucose detection in biological fluids, such as tears, with high sensitivity and without the nonspecific binding of impurities.

## 4. Conclusions

In this study, a glucose-responsive hydrogel electrode was developed for a highly sensitive and biocompatible glucose sensor based on the principle of FETs. HEMA/VPBA copolymerized hydrogels were optimized by varying the mixing ratio of their components. The optimized hydrogel FET showed glucose responsivity with high sensitivity and a molecular sieve effect preventing the nonspecific adsorption of albumin. Such a glucose-responsive electrode composed of soft materials is expected to be applied in wearable devices to detect glucose in biological fluids such as sweat, saliva, and tears. Additionally, a platform based on the principle of the FET biosensor is expected to be suitable for the highly sensitive detection of biological fluids with a low glucose concentration. The hydrogel-based glucose transistor may be combined with another device for controlling insulin release in the future, because hydrogels can be utilized to improve the biocompatibility of the glucose transistor.

## Disclosure statement

The authors declare no competing financial interest.

## Supplemental data

The supplemental material for this paper is available online at http://dx.doi//10.1080/14686996.2016.1257344.

## Funding

This study was supported by Program for Creating STart-ups from Advanced Research and Technology (START program) promoted by Ministry of Education, Culture, Sports, Science and Technology, Japan.

## Supplementary Material

100516_supplemental_material_sakata_STAM.docx
