# Supplementary material for: Glucose-responsive hydrogel electrode for biocompatible glucose transistor
Source: Sci Technol Adv Mater. 2017 Jan 9;18(1):26–33. doi: 10.1080/14686996.2016.1257344 (PMC5256429; doi:10.1080/14686996.2016.1257344)
Supplement: 100516_supplemental_material_sakata_STAM.docx [file tsta_a_1257344_sm4863.docx]

**Supplemental material**

 **Figure S1** Cross-sectional scanning electron microscopy (SEM) image of a hydrogel-coated Au/glass substrate. The fabricated hydrogel on a Au substrate was dried and observed by SEM (JSM-7500FA, JEOL Co., Ltd., Japan) at a voltage of 5.0 kV. The hydrogel contained 15% wt/wt solid content and 0.5% wt/wt VPBA. Scale bar: 1 μm.
